# Opportunities for building lifelong resilience and improving mental health for adolescents living with HIV

**DOI:** 10.1002/jia2.26377

**Published:** 2024-10-09

**Authors:** Wipaporn Natalie Songtaweesin, Paul Thisayakorn, Renata Arrington‐Sanders, Caroline Foster, Thanyawee Puthanakit

**Affiliations:** ^1^ School of Global Health, Faculty of Medicine Chulalongkorn University Bangkok Thailand; ^2^ Center of Excellence for Pediatric Infectious Diseases and Vaccines, Faculty of Medicine Chulalongkorn University Bangkok Thailand; ^3^ Department of Pediatrics, Faculty of Medicine Chulalongkorn University Bangkok Thailand; ^4^ Department of Psychiatry, Faculty of Medicine Chulalongkorn University Bangkok Thailand; ^5^ The Children's Hospital of Philadelphia, Perelman School of Medicine University of Pennsylvania Philadelphia Pennsylvania USA; ^6^ Imperial College Healthcare NHS Trust St Mary's Hospital London UK

1

Many children living with perinatally acquired HIV have now survived to adolescence/early adulthood. They are joined by those who acquired HIV as adolescents, with those aged 15−24 years representing the largest proportion of new HIV diagnoses globally [1]. Adolescence sees rapid cognitive, psychosocial, emotional and sexual development that can be associated with the onset of mental health disorders. These challenges can impact the development of resilience, which represents the social and emotional skills, attributes and habits that facilitate the overcoming of difficulties [2]. It is critically important to tailor service delivery that builds up positive mental health and resilience for adolescents living with HIV. However, mental healthcare provision has been hampered by restricted healthcare budgets, limited trained personnel and mental health and HIV stigma [3, 4]. This Viewpoint seeks to describe the intersection between adolescent development and mental health, and advocates for implementation of integrated mental healthcare delivery for adolescents living with HIV.

Adolescence refers to both an age range (10−19 years) and a life stage of complex development [5, 6]. Identity exploration and transition towards independence occur, with brain maturation completing in the late 20s [6]. Common challenges affecting adolescent health are related to the misalignment of different developmental domains that can result in risk‐taking behaviours, caregiver‐child conflicts and exploration of limits. For example, biological sexual maturation ahead of cognitive maturation can increase the likelihood of engagement in high‐risk sexual activity, which can result in both individual harm and conflict with caregivers.

Mental health is the state of wellbeing that enables people to cope with life stressors, discover oneself, and effectively function in and contribute to their community [2]. Three‐quarters of all mental health disorders start by adolescence [2, 7, 8]. HIV is a critical co‐factor in the evolution of mental health disorders, which occur in up to a quarter of adolescents with HIV [9]. Adolescents living with perinatally acquired HIV have grown up in a family affected by HIV and may be disproportionately impacted by adverse childhood experiences, including bereavement, poverty and migration or displacement [8, 10]. They are at risk for HIV‐related neurocognitive impacts from infancy, including HIV encephalopathy and opportunistic infections of central and peripheral nervous systems [8]. They have higher rates of mental health disorders such as anxiety and depression, with a possible increased risk of psychosis compared to age‐matched peers [11]. Those who acquire HIV during adolescence avoid the neurodevelopmental impact of HIV in infancy/early childhood, and consequently may have better physical and neurological development. However, HIV acquisition in adolescence is associated with childhood adversity, including socio‐economic hardship, failure to complete secondary education, lack of family support and orphanhood [12]. In addition, many are from key population groups (e.g. males who have sex with males, transgender people) and have frequently experienced gender identity‐related stigma and discrimination.

All adolescents living with HIV experience HIV‐related stigma, disclosure‐related anxiety and fear of rejection. The interaction between the above social, physical and mental health burdens faced by adolescents living with HIV are additive, resulting in “syndemics” that further contribute to disproportionate disease burdens [13]. An example of these syndemic interactions is that mental health disorders can lead to poorer adherence to antiretroviral therapy resulting in virological failure, which in turn can lead to HIV‐associated neuroinflammation, all of which can exacerbate mental health disorders [10].

Implementing tailored mental health service delivery for adolescents living with HIV should consider both the individual and the context in which they live. For the individual, an understanding of the psychosocial and cognitive changes taking place in adolescence ensures that interventions are developmentally appropriate [6]. The context is readily conceptualized within a social ecological model, which acknowledges the intersecting elements impacting health behaviours and outcomes, including individual, interpersonal, environmental and macrosocial factors [1].

A variety of evidence‐based interventions that foster resilience and improve mental health are available for implementation in different settings that address both individual and contextual factors (Table 1). Cognitive‐behavioural therapy can support mental health management, including addressing internalized stigma. Peer‐led mental health support leverages the importance adolescents give to peers, while family‐strengthening interventions are based on the critical role families play in facilitating adolescent mental wellbeing. Integration of mental health services into existing HIV services builds on established patient‐provider rapport and improves access to mental healthcare. An example is the collaborative care model (CCM), where primary care teams are guided by specialists to deliver what previously were referral‐level clinical services through task‐shifting. In Thailand, CCM used in primary HIV care to deliver adolescent mental healthcare has resulted in a 63% reduction in psychiatric referrals, reflecting its feasibility in resource‐limited settings [14].

**Table 1:** Selection of evidence‐based interventions for adolescent mental health^a^

Intervention	Description	Examples in practice
Cognitive‐behavioural therapy (CBT)	Education on the link between thoughts, feelings and behaviours; guidance to interrupt unhelpful thinking cycles and replace with helpful thoughts	Mindfulness stress reduction programme Use of mindfulness to increase present moment awareness, manage stress Management of stigma, enhancement of self‐concept
Peer‐led support	Counselling delivered by adolescents who have training in communication skills, mental health and HIV knowledge to provide mental healthcare and linkage to further services if needed	Community adolescent treatment supporters Youth training on adherence and psychosocial support counselling Weekly home visits to provide adherence and psychosocial support
Family‐strengthening interventions	Education on increasing understanding of psychosocial needs of family members, health knowledge and communication skills	Family strengthening intervention for HIV Promotion of parent‐child relationships in families with adolescents living with HIV Manualized, delivered by bachelor‐level counsellors Eight sessions of 90‐minute home‐visits
Integration of mental health and psychosocial support with HIV services	Mental health services are incorporated into the functioning of another established service	Collaborative care model: primary care teams guided by specialists to deliver what previously were referral‐level clinical services through task‐shifting Benefits: Allows teams to leverage existing patient‐provider rapport Reduces the need for psychiatrist‐managed care Builds primary team capacity Cost‐effective

^a^ Adapted from 1) Bhana A et al. JIAS. 2021;24 (Suppl 2):e25713, and 2) Integrating psychosocial interventions and support into HIV services for adolescents and young adults: technical brief. Geneva: World Health Organization; 2023 [9, 15].

Implementation of adolescent services can employ simple, standardized tools, such as the patient health questionnaire (PHQ‐9) to screen for depression and suicidality, and SSHADESS, a holistic strengths‐based psychosocial assessment which asks about Strengths, School, Home, Activities, Drugs, Emotions, Sexuality and Safety. A caring and non‐judgemental approach is critical in creating a client‐centred and safe space for adolescents.

In line with global calls to “put people first in the global HIV response,” we advocate for adolescent‐empowering care structures that promote the development of lifelong mental health and resilience for adolescents living with HIV. Evidence‐based interventions can only be put into practice with supporting policies and reimbursement systems. This includes embracing task‐shifting that builds capacity to manage mental health for adolescents living with HIV outside of existing specialist structures, and conducting implementation research to strengthen such policies.

The WHO 2022 World Mental Health Report acknowledged that “no country is expected to fulfill every implementation option in the global action plan,” but goes on to say that “every country can make meaningful progress towards better mental health for its population” [2]. We must take action during the period of adolescence to address the disproportionate burden of mental health disorders experienced by those living with HIV if we are to build lifelong resilience and mental wellbeing that allows this vulnerable group to fulfil their enormous potential in adult life.

## COMPETING INTERESTS

The authors report no conflicts of interest.

## AUTHORS’ CONTRIBUTIONS

All authors contributed to the writing and approval of the manuscript.
